# Preemptive analgesia-related gene and protein expression in third molar surgeries under non steroidal anti-inflammatory drug protocols: A PROSPERO-registered systematic review of clinical studies

**DOI:** 10.4317/medoral.22576

**Published:** 2018-11-21

**Authors:** Assis-Filipe Medeiros-Albuquerque, Carline-Maria Sampaio-Melo, Eduardo-Costa Studart-Soares, Thyciana Rodrigues-Ribeiro, Cristiane-Sá Roriz-Fonteles, Karuza-Maria Alves-Pereira, Daniel-Almeida Ferreira-Barbosa, Paulo-Goberlânio de-Barros-Silva, Fábio-Wildson Gurgel-Costa

**Affiliations:** 1 Post-graduate Program in Dentistry, School of Dentistry, Federal University of Ceará, Ceará, Fortaleza, Brazil; and Division of Oral Surgery, School of Dentistry, Fortaleza University (UNIFOR), Ceará, Fortaleza, Brazil; 2 Student in Dentistry, School of Dentistry, Fortaleza University (UNIFOR), Ceará, Fortaleza, Brazil; 3 Post-graduate Program in Dentistry, School of Dentistry, Federal University of Ceará, Ceará, Fortaleza, Brazil

## Abstract

**Background:**

This study aimed to review translational studies focusing on third molar removal surgeries through a systematic analytical approach.

**Material and Methods:**

A PROSPERO-registered systematic review (CRD42017060455) was conducted following the PRISMA statement to summarize current knowledge on gene expression in third molar surgeries. A search was performed in PubMed’s Medline and Scopus databases, without date or language restrictions, using the logical expression {[(Third molar) OR (preemptive) OR (cyclooxygenase inhibitors) OR (acute inflammation) AND (gene expression)]}.

**Results:**

All studies included in the analysis evaluated gene expression in a third molar extraction model, using the preemptive analgesia methodology in seven investigations. The sample analyzed was obtained from gingival tissue biopsy (n=4), blood (n=1), transudate (n=1) and gingival tissue biopsy/transudate (n=1). There were differences with respect to evaluated genes, drug protocol, sample studied, and method for evaluating gene expression.

**Conclusions:**

Third molar surgeries were found to be associated with different COX-related gene expression patterns. Although inflammatory events following the surgical procedure are associated with COX isoforms, data from preemptive analgesia studies are scarce, especially from studies correlating gene expression and clinical parameters. In the future, from a clinical perspective, identifying the molecular targets of a drug based on individual gene expression may be helpful to delineate specific third molar, surgery-related, preemptive analgesia protocols.

** Key words:**Third molar, gene expression, preemptive analgesia, systematic review.

## Introduction

The evaluation of specific gene expression has been widely used as an important tool in studies in the field of dentistry, including translational investigations to determine diagnostic approaches, as well as to evaluate pharmacology-based drug protocols commonly utilized in clinical situations (1,2). In addition, genetic analyses through observation of messenger RNA (mRNA) expression or the measurement of specific mediators such as cytokines have produced important findings that support a relationship between tissue damage, degree of inflammatory response, and onset of clinical related events such as pain and edema in surgical procedures for the removal of third molars (3-5).

Removal of third molars is an invasive procedure capable of triggering varying levels of pain and other related inflammatory events, which can significantly affect the patient’s quality of life (6). These factors have contributed to the routine use of third molar surgery as a useful clinical model to analyse the efficacy of conventional prescribed analgesics and anti-inflammatory drugs in order to minimize the effects of inflammation arising from surgical intervention until the postoperative period (7,8). As pain and edema are expected following the surgical procedure, it is predicted that these events, elicited by inflammation, would be correlated with gene-related increases of key pro-inflammatory cytokines released at the site of the injury, e.g. interleukin 1 beta (IL-1β) and tumor necrosis factor alpha (TNF-α). Indeed, these markers have been shown to have significantly increased levels after the extraction of third molars (3) and are directly linked to cyclooxygenase isoform gene expression during the inflammatory process (9).

Cyclooxygenase gene evaluation has attracted substantial interest due to its value in both the laboratory setting and clinical situations since the routine use of selective and non-selective COX-2 non-steroidal anti-inflammatory drugs (NSAIDs) may be associated with different levels of COX expression, facilitating the testing of these medicines for relief of third molar-related inflammatory symptoms (3,10). The use of methods for the quantification of COX expression following clinical procedures under pharmacological analgesic and anti-inflammatory protocols should help to explain the influence of these drugs on pain variables and lead to significant improvements in treatment (10-12).

Other genes besides COX isoforms have been studied in the field of third molar surgery as target genes during the inflammatory process (2). Recently, certain authors performed quantitative analysis of gene expression in translational researches evaluating the effect of preoperative administration of different NSAIDs on the severity of clinical events related to inflammation in patients who underwent the surgical removal of third molars (12,13). Although the quantitative real-time polymerase chain reaction (RT-qPCR) is considered a standard methodology, widely used in experimental investigations (14), other methodologies have been proposed for evaluating gene expression in third molar surgeries in order to find correlations with postoperative clinical symptoms, such as pain and edema (11,13). However, an overview of these studies by means of a systematic analysis was not published to date. Systematic reviews are important approaches designed to investigate specific issues of scientific interest using clear, well defined, and rigorous methods (15). These studies characteristically involve a meticulous and comprehensive plan and search strategy, defined a priori, that aims to reduce bias by identifying, appraising, and synthesizing all pertinent studies on a certain topic (16,17).

The scientific value of systematic reviews depends on several factors. Despite a standardized approach, some articles have historically suffered methodological failures in relation to the structuring of the research. Consequently, both the “Preferred Reporting Items for Systematic Review and Meta-Analysis (PRISMA)” criteria, available since 2009 (18), as well as the register of these studies at the web-based platform, the “International prospective registry of systematic reviews (PROSPERO)”, accessible since 2011, have been adopted here in order to minimize inconsistencies and bias during the review process (15,19). The importance of obtaining adequate, objective data regarding COX gene expression in translational studies dictated the present PROSPERO-registered systematic review, based on standardized methodology and following the PRISMA guide recommendations.

## Material and Methods

-Protocol and Registration

A systematic review was conducted to summarize current knowledge concerning gene expression recorded in clinical studies involving the preemptive use of NSAIDs in third molar surgeries. This systematic review was registered in the PROSPERO database and conducted following the PRISMA statements under the number 42017060455.

-Information Sources and Search Strategy

The PICO strategy (Patient/Population: patients; Intervention: preemptive analgesia for third molar removal; Comparison: gene expression; Outcome: studied variables) was used to formulate the initial question to be answered by this systematic review: “Is there evidence of variation of gene expression in patients who underwent preemptive analgesia with NSAIDs during third molar surgeries?” 

In order to perform the search strategy, PubMed’s Medline, Scopus, and SciencDirect were used as electronic databases to retrieve articles without date or language limits. The systematic review was conducted on April 10, 2017, and the computer network of the Federal University of Ceará (Brazil), School of Dentistry was used to perform the electronic data search. The algorithm used was: {[(Third molar) OR (preemptive) OR (cyclooxygenase inhibitors) OR (acute inflammation) AND (gene expression)]}.

Other sources were also used to include additional articles. A manual search of related journals was performed, including: 1) Medicina oral patología oral y cirugia bucal, 2) The British journal of oral & maxillofacial surgery, 3) International journal of oral and maxillofacial surgery, 4) The journal of craniofacial surgery, 5) Journal of cranio-maxillo-facial surgery, 6) Journal of maxillofacial and oral surgery, 7) Journal of oral and maxillofacial surgery, 8) Anesthesia and analgesia, 9) Prostaglandins, leukotrienes, and essential fatty acids, 10) Oral surgery, oral medicine, oral pathology and oral radiology, 11) Clinical pharmacology & therapeutics, 12) British journal of pharmacology, 13) The clinical journal of pain, 14) British journal of rheumatology, 15) European journal of pharmacology, 16) Pain, 17) Inflammation research, 18) The journal of pain, and 19) Anesthesiology. Reference lists obtained from the identified articles and relevant reviews on the subject also were checked for possible additional studies.

-Eligibility criteria

The inclusion criteria adopted in this review were: articles without language or year of publication restrictions, clinical studies involving gene expression in oral surgery, and studies involving human beings. Exclusion criteria, items not considered eligible for inclusion, were: case reports, case series, literature reviews, and editor’s notes.

-Study selection and data collection process 

A two-phase selection of the articles was conducted. In Phase 1, two independent researchers (AFMA and CMSP) determined eligibility by reading titles and abstracts of each identified study; subsequently, some articles found in different databases were excluded on the basis of duplication. In Phase 2, the full text of eligible articles was assessed following the inclusion criteria. Any reviewer disagreements were resolved by consensus with a third researcher (FWGC).

The researchers independently extracted the data using previously established criteria. Each selected study was analyzed and the following variables adopted for the systematic review were summarized when available: study origin, number of patients, sex, age, use of preemptive analgesia therapy, studied drugs, type of material collected for gene expression analysis, evaluation time, and quantitative data on the studied genes.

-Risk of bias in individual studies

The methodological validity of selected studies was assessed by two independent reviewers (AFMA and CMSP) using the Joanna Briggs Institute Meta Analysis of Statistics Assessment and Review Instrument (JBI-MAStARI) as previously reported (20). The reviewers independently scored each data item as “yes”, “no”, “unclear” or “not applicable” and assessed the quality of each included study. A third author (FWGC) resolved any disagreement between the reviewers through discussion. Risk of bias was categorized as high (up to 49% scored “yes”), moderate (50–69% scored “yes”), and low (more than 70% scored “yes”).

-Synthesis of results

Data were imported into an Excel (Microsoft Corporation, Redmond, WA) spreadsheet which was used to calculate relative and absolute data frequencies.

## Results

-Study selection

**Figure 1:**
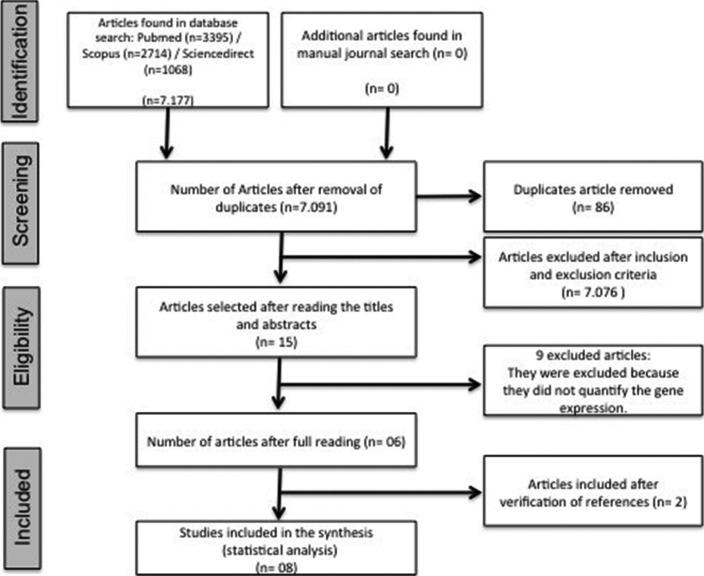
Flowchart showing the eligibility criteria for article selection adopted in the present study.

The article selection process is presented in Figure 1. The search strategy identified an initial 7,177 articles, of which 86 studies were found in more than one database (duplicates) and were removed. Of the remaining articles, 7,076 were excluded because they did not discuss the topics adopted for investigation in the present study. Manual searches in related journals did not result in addition studies and the 15 remaining qualifying articles were completely read. Nine of these studies were excluded because they did not meet the eligibility criteria since they did not evaluate gene expression in third molar surgeries. Searches of the reference lists from the selected studies rendered two additional articles. Consequently, seven articles were ultimately evaluated in this review.

-Study characteristics

All of the selected studies originated from North America (USA), representing a total of 929 patients, with a predominance of males (69%), females (31%). The subjects were mainly adults, with ages ranging from 16 to 66 years and an approximate mean age of 25 years ( Table 1).

**Table 1:**
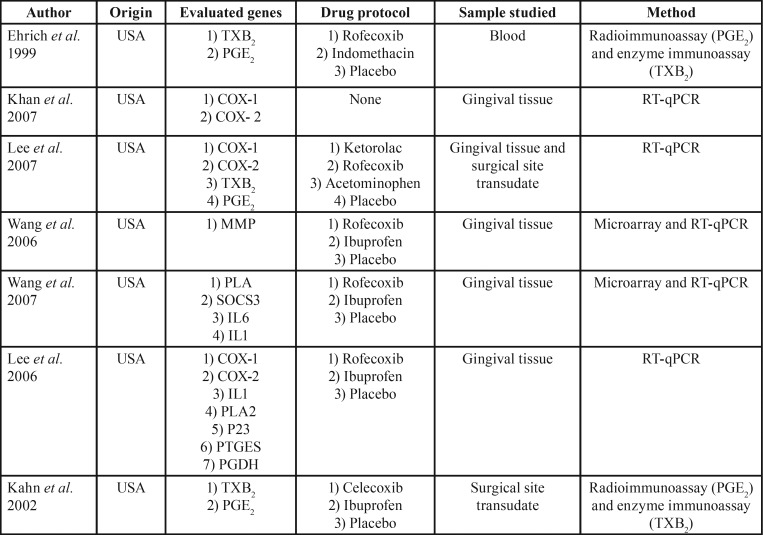
Characterization of the selected studies regarding year of publication, origin, evaluated genes, drug protocol, sample studied, and performed test.

All of the articles analyzed gene expression in patients undergoing third molar surgery. The samples evaluated for this purpose were gingival tissue removed during the surgical procedure in five studies, n=722 patients (2,4,5,20,21), followed by blood analysis in one investigation, n=104 patients (22), and gingival exudate analysis in another study, n=103 patients (13).

-Risk of bias within studies

**Figure 2:**
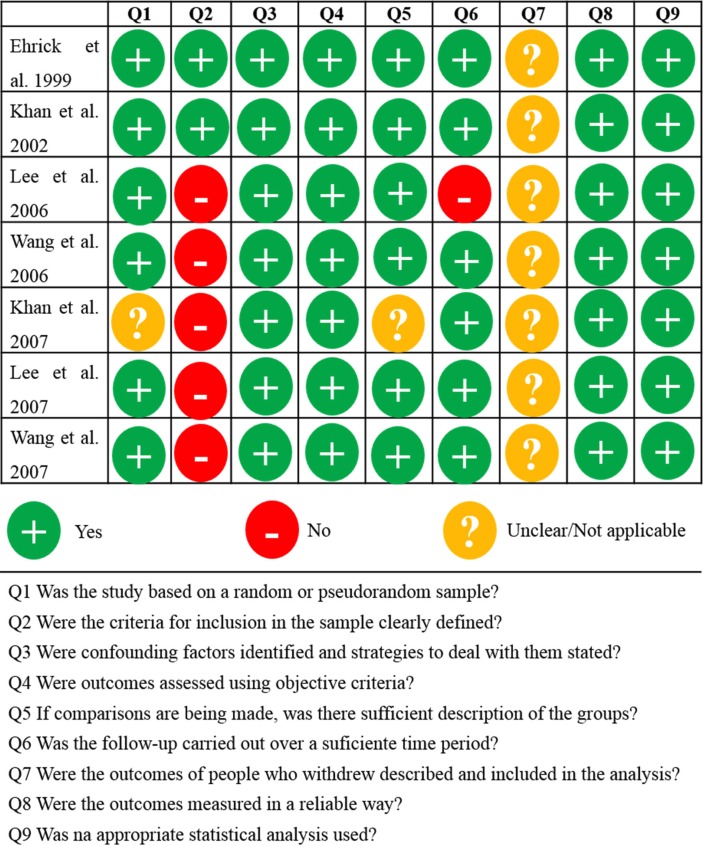
Critical appraisal checklist summary for randomized control/pseudo-randomized trials (JBI-MAStARI).

As shown in Figure 2, the mean percentage of “yes” scores was 76.2±11.9, ranging from 77.8% to 88.9%. The risk of bias within studies was considered moderate in two studies and low in five studies.

Results of individual studies

There was a preponderance of COX-2 selective NSAID treatments among the articles published in the field of the preemptive analgesia. The following drugs were used: rofecoxib, n=5 articles (2,4,21–23); ibuprofen,n=5 articles (2,13,21–23); ketorolac, n=1 article (4); acetaminophen, n=1 article (4); indomethacin, n=1 article (23); celecoxib, n=1 article (13), and placebo, n=6 articles (2,4,13,21–23). In all of these studies, methodologies were applied in order to obtain data about COX gene expression. A variety of methods were used and these are summarized in  Table 1; three studies quantified gene expression by using RT-qPCR analysis and the remaining studies performed an indirect evaluation of the COX expression by analyzing related genes: thromboxane B2 (TXB2) (4,13,23), prostaglandin E2 (PGE2) (4,13,23), matrix metalloproteinase (MMP) (21), phospholipase A2 (PLA), suppressors of cytokine signaling 3 (SOCS), and interleukins 1 and 6 (IL-1 and IL-6, respectively) (2,22).

Pain was the common clinical parameter evaluated as a primary outcome reflecting the inflammatory events following surgical removal of third molars in these studies. Among these studies, pain was measured by means of visual analog scales (VAS) in four investigations (2,4,13,23), and the others only included a laboratory analysis without proper clinical observation. Pain was evaluated over different study periods or at certain time points, such as 0–6 h (n=1), 0–180 min (n=1), 2–4 h and 48 h (n=1), or 24 h (n=1) postoperatively. In addition, VAS was used in two articles and scores ranging from 1 to 4 (mild, moderate, grave, and severe respectively) were employed in the other studies.

When the pharmacological class of the studied NSAIDs was evaluated with respect to COX gene expression (2,4,21,22), it was observed that coxibs (rofecoxib and celecoxib) presented a significant selectivity related to COX-2, ranging from 5 to 500 times greater than other non-selective NSAIDs (ibuprofen, acetaminophen, and indomethacin) or placebo, being evaluated by RT-qPCR or by analyzing genes that display influence on COX-1 and COX-2 gene expressions, such as TXB2, PGE2, and MMP.  Table 2 presents the principal results found in the studies.

**Table 2:**
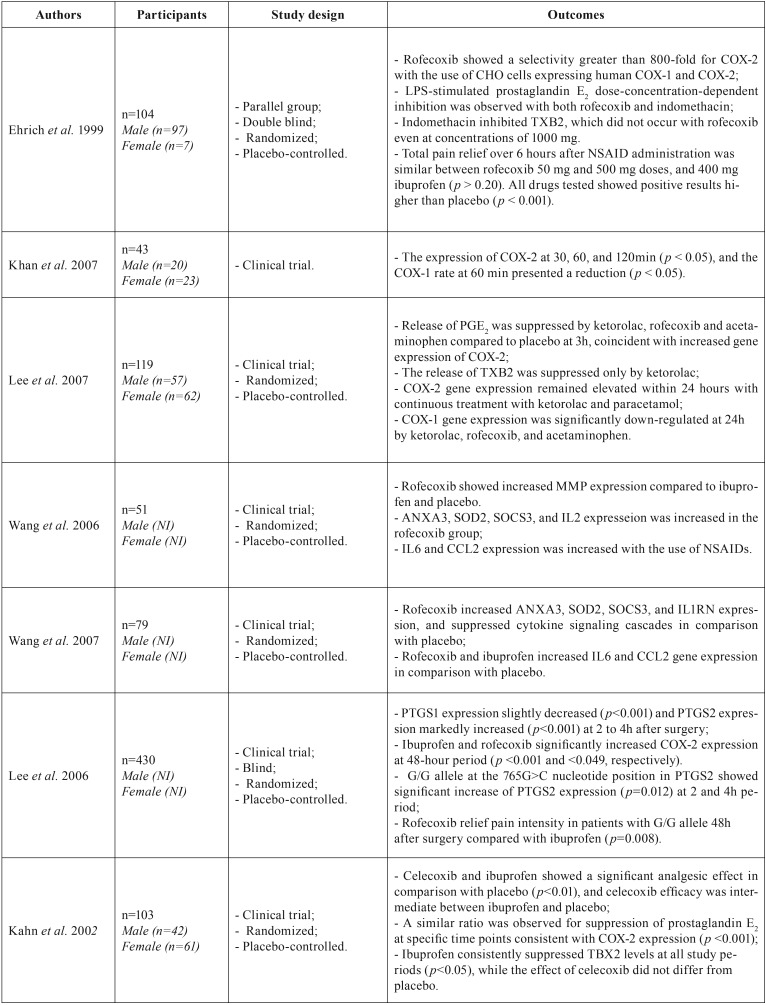
Characterization of the selected studies according to the sample number, study design, outcomes, and conclusion.

## Discussion

Third molar surgery is a routine clinical procedure performed at dental practices, and it has been the subject of studies evaluating the use of preoperative medications (NSAIDs) to minimize inflammatory events observed during the postoperative period (7). In fact, this clinical model has been widely reproduced in translational researches aiming to evaluate protocols of medicines since 1976, when its validation in pharmacological studies was provided (24). The present systematic review based on the PRISMA methodology reported relevant data related to of oral surgery with evaluation of gene expression following removal of maxillary and mandibular third molars.

A considerable variety of methodologies were found to have been used for clinical and laboratory purposes, including the gene studied, the preemptive analgesic medication, the type of material collected for gene expression analysis, study periods, and the method for analyzing gene-related mRNA. Since several methods to evaluate gene expression of COX or related genes was performed among the critically reviewed articles some difficulty was encountered in obtaining a standardized and reliable analysis. Of the eligible studies, there were three investigations that directly evaluated the COX genes (2,4,5), while the remaining studies used an indirect method to provide the evaluation. These articles analyzed TXB2 and PGE2 (4,13,23), MMP (20), or a set of genes including PLA, SOCS3, and IL-6 (21,22).

These genes are involved in the inflammatory response, which is mediated by prostaglandins produced via cell membrane phospholipid-related arachidonic acid metabolism. It can be triggered by two COX pathways. COX-1 is the isoform constitutively expressed which is involved in the regulation and homeostasis of physiological processes, but is also active during the onset of acute inflammation. COX-2 is an inducible isoform produced in inflammatory and infectious processes, showing a direct relation with the increased production of pro-inflammatory prostaglandins and cytokines such as PGE2, TNF-α, and IL-1β (3,9,22). PGE2 is released into inflamed tissues in order to sensitize afferent nerve fiber terminals, increasing the nociceptive process and evoking hyperalgesia (13,23), while TNF-α exerts a remarkable range of effects, including activating lymphocytes, stimulating the synthesis of other proinflammatory cytokines such as IL-1β and IL-6, and triggering the production of prostaglandins. IL-1β also sensitizes nociceptors and causes hyperalgesia, acting to promote pain pathophysiology (3). mRNA related to COX-1 expression has a half-life of about 12–15 h while COX-2 mRNA has a shorter half-life of less than 3.5 h, suggesting an intrinsic temporal link between tissue injury, COX-2 expression, and increased PGE2 levels compared to COX-1 expression, which is responsible for coagulation and directly linked to COX-1 activity (4,13,23). In addition, matrix metalloproteinases (MMP) play an important role in the inflammation and is regulated by PGE2, serving as a reference for COX-2 (21). Other genes that are also related to inflammation but scarcely studied are IL-6, SOCS3 and PLA, which may increase with inflammation, serving as indicative regulatory parameters for COX levels locally (22).

Ehrich *et al.* (23) evaluated COX isoform expression indirectly (TXB2 for COX-1 and PGE2 for COX-2) from blood samples and showed that rofecoxib was a potent selective COX-2 NSAID, exhibiting about 800 times greater selectivity for COX-2 than COX-1, and it exhibited about 1000 times greater COX-2 selectivity in comparison with a non-selective NSAID (indomethacin). Furthermore, as previously confirmed by Lee *et al.* (4) in a study with gingival tissue samples, it was observed that TXB levels did not change significantly. In addition, it was demonstrated that COX-1-related activity in the placebo and testing groups (rofecoxib and acetaminophen) showed no significant differences. The preoperative administration of these NSAIDs to individuals undergoing third molar surgeries resulted in suppression of PGE2 levels compared to the placebo group, highlighting the COX-2 selectivity pattern.

According to Kahn *et al.* (13), TXB2 gene expression is specifically related to the COX-1 isoform, while PGE2 gene expression was highly associated with COX-1 levels in a 60-min period following the onset of the inflammatory process and to COX-2 levels. In the latter study, COX-2 selectivity provided by celecoxib administered preemptively resulted in PGE2 gene suppression without alteration of TXB2 levels during the postoperative period beyond 60 min. Ibuprofen, however, suppressed both PGE2 and TXB2 over both evaluated study periods, which is consistent with its dual COX-1/COX-2 inhibitory effects.

Clinically, several trials have shown that NSAIDs ameliorate the symptoms associated with third molar surgeries (3,6,7,10). On the basis of inflammation assessments, MMP family-related genes have been linked with a decrease in the severity of the inflammatory process when COX-2 selective drugs are prescribed to patients that underwent surgical removal. Wang *et al.* (21) showed that in samples of gingival tissue, MMPs play an essential role in acute inflammatory injury and their activity is regulated by COX-2 mediated PGE2 release. They reported a significant increase in MMP expression in a clinical study using rofecoxib in comparison to ibuprofen and placebo, which may contribute to rofecoxib-associated adverse effects: interference with the resolution of inflammation and onset of undesirable effects. In addition to the MMP results, other genes mediated by COX-2 expression and associated with the occurrence of inflammatory events i.e., the arachidonic acid pathway, apoptosis/angiogenesis, cell adhesion, and signal transduction were also analyzed (21). Wang *et al.* (22) also observed that gene expression of ANXA3 (annexin 3; involved in the regulation of inflammatory responses, cell differentiation and cytoskeletal protein interactions, associated with multiple human diseases); SOD2 (superoxide dismutase 2: expressed in the central nervous system in several inflammatory conditions); SOCS3 (suppressor of cytokine signalin 3, which regulates the signaling of cytokines or hormones, modulating the outcome of autoimmune infections and diseases, as well as the underlying mechanisms); and IL1RN (IL1 receptor antagonist, associated with several markers of systemic inflammation) were increased in the group treated with rofecoxib. These were predictable results, since these genes are related to the inhibition of phospholipase A2 after local trauma and decrease in cytokine signaling pathways. In addition, groups treated with rofecoxib or ibuprofen showed an increase in gene expression of the inflammatory mediators IL-6 (a cytokine involved in both the inflammatory and infection responses, and in the regulation of metabolic, regenerative, and neural processes), and CCL2, chemokine (C-C motif) ligand 2, (which is involved in neuroinflammatory processes and is present at the sites of tooth eruption and bone degradation), after surgical trauma when compared to the placebo. These results emphasize that COX selectivity is involved not only in anti-inflammatory effects but also in the increase in pro-inflammatory cytokine levels and hence may play a role during the inflammatory process in local injuries, such as the third molar removal (22).

Regarding COX gene expression over time, Kahn *et al.* (5) performed a quantitative analysis of COX-1 and COX-2 isoforms in a clinical study of third molar extraction. These authors observed that all samples used to evaluate COX-2 gene expression showed a very weak band at baseline assessment (51%). However, there was a significant, progressive increase in COX-2 expression at 30, 60, and 120 min after surgery. When COX-1 expression was measured, a slight decrease was detected at 30 min, a considerable reduction at 60 min, and a significant reduction compared to baseline at 120 min. In fact, these findings related to the expression of COX-2 were expected, considering that no preemptive NSAIDs were used and that only the normal inflammatory process was triggered by surgical extraction of a third molar. In the case of the COX-1 results, other studies that indirectly evaluated its expression supported these findings (4,13,21,22). Another study that quantitatively described COX-1 and COX 2 gene expression was performed by Lee *et al.* (2), in which an increase in COX-2 and a decrease in COX-1 were observed at 2–4 hours postoperatively, returning to pre-surgical values at 48 h after surgery. These findings suggest that acute injury resulting in inflammatory processes stimulates increased gene expression of COX-2 and transient inhibition of COX-1 expression. There was also a slight increase in the expression of IL-1β (2–4 h), PLA2 (2–4 h and 48 h) and a decrease in PTGH levels (an enzyme encoded to degrade prostaglandins) over time (2).

Postoperative pain was assessed in four translational studies that used a laboratory analysis in order to asses COX gene expression following third molar surgery (2,4,13,23). In the study performed by Ehrich *et al.* (23), the analgesic efficacies of rofecoxib and ibuprofen were postoperatively evaluated and significant reduction in pain perception in both of groups was found in comparison to the placebo group; however, there was no statistically significant difference between the NSAIDs. The time required for pain relief did not differ between the groups, but it was statistically significant in comparison to the placebo. The use of rescue analgesia during the two-hour postoperative period was reported by 75% of patients treated with placebo, whilst test groups (rofecoxib and ibuprofen) comprised 25% that received rescue drugs up to six hours after the surgical removal of the third molar, highlighting the efficacy of the NSAIDs in relieving pain postoperatively.

In the study of Lee *et al.* (4) that evaluated the preemptive analgesic efficacy of rofecoxib, acetaminophen, and ketorolac compared with the placebo, there was a gradual increase in pain scores over the first three hours showing no statistically significant differences between the rofecoxib and acetaminophen groups or in comparison with the placebo group; however, preoperative ketorolac use delivered the best results with respect to pain relief over a two-hour study period together with rescue medication intake and the cumulative effect of pain scores over the time in comparison with placebo. In contrast to acetaminophen, rofecoxib showed a statistically significant reduction of pain scores after two hours following the surgical procedure. The authors of this study also pointed that the results were consistent in relation to gene expression of TXB2 and PGE2. Lee *et al.* (2) showed that gene expression of COX isoforms was not the only factor supporting the observed analgesic efficacy of rofecoxib and ibuprofen preemptively used. These authors investigated COX gene polymorphism and showed that in patients with the homozygous alleles for COX-2 (G/G) there was a significant reduction in pain 48 h after administration of rofecoxib in comparison with that after administration of ibuprofen. In individuals with reduced expressivity of alleles (homozygous and heterozygous, C/C and G/C respectively), the opposite effect on pain relief at 48 h postoperative was observed and better pain scores were obtained with ibuprofen. Thus, these findings reinforce the importance of gene polymorphism in determining the efficacy of preemptive analgesic medication following third molar surgeries.

In another investigation performed by Kahn *et al.* (13), it was shown that celecoxib and ibuprofen delivered better results in relieving pain postoperatively than the placebo group, which presented increasing pain intensity over the studied periods. However, celecoxib did not differ from placebo regarding pain relief at certain times (120, 180, and 240 minutes). In contrast, ibuprofen resulted in pain scores consistently reducing over time.

## Conclusions

In summary, third molar surgeries were associated with different COX-related gene expression patterns. Although inflammatory events following the surgical procedure are associated with COX isoforms, it was found that data from preemptive analgesia studies are scarce, especially those correlating gene expression with clinical parameters. The present findings were revealing in relation to the effect of selective and non-selective NSAIDs administered preoperatively with the aim of controlling the postoperative pain level. In the future, from a clinical perspective, identifying the molecular targets of a drug based on individual gene expression may be helpful in the delineation of specific, third molar surgery-related, preemptive analgesia protocols.
